# Comparison of cardiovascular disease risk in women with and without breast cancer: secondary data analysis with the 2014–2018 korean national health and nutrition examination survey

**DOI:** 10.1186/s12889-023-16063-2

**Published:** 2023-06-15

**Authors:** Seongmi Choi, Na-Jin Park, Mihui Kim, Kijun Song, JiYeon Choi

**Affiliations:** 1 College of Nursing and Brain Korea 21 FOUR Project, Yonsei University, Seoul, 03722 South Korea; 2 University of Pittsburgh School of Nursing, Pittsburgh, PA 15261 USA; 3 Mo-Im Kim Nursing Research Institute, Yonsei University College of Nursing, Seoul, 03722 South Korea; 4 Department of Nursing Science, Jeonju University, Jeonju-si, 55069 Jeollabuk-do South Korea

**Keywords:** Breast cancer, Cardiovascular disease, Framingham risk score, Waist-to-height ratio

## Abstract

**Background:**

Aging breast cancer survivors may be at an elevated risk of cardiovascular disease (CVD), but little is known about CVD risk assessment and breast cancer in Korean women. We hypothesized that Korean breast cancer survivors would have higher risks of future CVD within the next 10 years (i.e., Framingham Risk Score [FRS]) than women without cancer.

**Objectives:**

(1) To compare FRS-based CVD risks in women with and without breast cancer based on propensity score matching; and (2) To explore adiposity-related measures in relation to FRS in Korean women with breast cancer.

**Methods:**

Using the cross-sectional data from the 2014–2018 Korean National Health and National Survey (KNHANES), we identified 136 women with breast cancer aged 30–74 years who had no other cancer and no CVD. The comparison group of 544 women with no cancer were selected by 1:4 nearest-neighbor propensity score matching based on breast cancer diagnosis. CVD risk was assessed by FRS based on multiple traditional risk factors (e.g., cholesterol, blood pressure, diabetes, and smoking). Adiposity was measured by physical examination, including body mass index (BMI) and waist-to-height ratio (WHtR). Physical activity and health behaviors were assessed by self-reports.

**Results:**

Women with breast cancer (mean age of 57 years) had similar FRS levels at a low-risk category (< 10%) to women with no cancer (4.9% vs. 5.5%). Breast cancer survivors (mean 8.5 survival years) presented at significantly lower levels of total cholesterol, BMI, and WHtR (all *p* values < 0.05) than their counterpart. Within the breast cancer group, WHtR ≥ 0.5 was associated with higher FRS, compared to WHtR < 0.5. FRS was not different by survival < 5 years or ≥ 5 years after breast cancer diagnosis.

**Conclusions:**

FRS-based CVD risks were not different in Korean, mostly postmenopausal, women by breast cancer status. Whereas breast cancer survivors had even lower levels of lipid and adiposity measures than women without cancer, those values indicating borderline cardiometabolic risk suggest continued screening and management efforts for these aging women. Future studies are needed to examine longitudinal trajectories of CVD risk factors and CVD outcomes among Korean breast cancer survivors.

## Introduction

Breast cancer is the most common cancer along with thyroid cancer among Korean women, particularly in their 40 and 50 s [1]. Due to advances in early detection and effective treatment, the incidence of early-stage breast cancer continues to rise with improved breast cancer-specific survival rates in Korea [2]. Women with breast cancer are expected to live longer than ever, leading to a growing number of breast cancer survivors who are also aging and at an elevated risk of cardiovascular disease (CVD) [3, 4].

Both breast cancer and CVD share multiple risk factors (e.g., age, adiposity, physical inactivity, and diabetes) [5, 6]. In combination with adverse cardiotoxic effects of treatment regimens (e.g., chemotherapy and radiotherapy), studies have shown that women with breast cancer were likely at an increased risk of CVD, during active treatment or decades after cancer treatment has ended [7, 8]. Following anti-cancer treatment, women with breast cancer also experience poor lifestyle changes, such as weight gain and physical inactivity, further exacerbating their risk of CVD [9–11]. Indeed, CVD morbidity and mortality are known to be increased about 5 to 7 years after breast cancer diagnosis, and CVD, not cancer itself, is the leading cause of death in older survivors [4, 5, 12, 13]. Independent of breast cancer treatment status, pre-existing and post-treatment CVD risk factors are strong predictors of CVD and all-cause mortality over time [13–20]. Incorporating proactive preventive strategies for CVD risk factors to the survivorship care plan would improve CVD and all-cause mortality outcomes in women with breast cancer. However, there is no consensus on standardized guidelines for CVD risk assessment and management specific to breast cancer survivors over their extended periods of survivorship [21].

Few observational studies for Koreans have reported a higher CVD risk in adult cancer survivors compared to adults with no cancer [12]. However, the two groups were compared without adjusting differences in their baseline characteristics (e.g., age, sex, education, income, etc.) [12]. For example, the adult cancer survivors were much older than non-cancer controls (mean age of 60 vs. 45 years). Moreover, none have focused on CVD risk assessment and breast cancer in Korean women. Using the Framingham Risk Score (FRS) based on multiple traditional risk factors (e.g., age, hypertension, lipids, smoking, and diabetes) [22], we hypothesized that breast cancer survivors would have a higher risk of future CVD event within the next 10 years (i.e., FRS) than women without cancer in Korea. Emerging evidence suggest that adipose tissue distribution (i.e., abdominal adiposity) is a better predictor of CVD death and overall mortality after breast cancer diagnosis, including among women with a normal body mass index (BMI) [9, 23, 24]. However, such relationships have not been validated for Korean women with breast cancer because current measures of adiposity do not reflect individual differences across gender, ethnic groups, or country [25].

Therefore, the objective of this study was to compare the FRS-based CVD risks in women with and without breast cancer using propensity score matching to minimize selection bias for baseline sociodemographic characteristics (e.g., education, income, residence area, and menopause). In addition, we explored abdominal adiposity-related measures, such as waist-to-height ratio (WHtR), in relation to FRS in Korean breast cancer survivors.

## Methods

### Data source

This study was the secondary data analyses using the Korean National Health and Nutrition Survey (KNHANES) data from 2014 to 2018. The KNHANES, conducted annually by the Korea Disease Control and Prevention Agency, is a nationwide cross-sectional survey that represents the general Korean population [26]. The KNHANES uses a stratified, multi-stage, clustered probability sampling design according to region size and demographic characteristics to select a representative sample of civilian, non-institutionalized South Koreans. To provide a broad perspective on health risk behaviors and indicators and chronic diseases, the survey combines two components: (1) health and dietary interviews (e.g., sociodemographic characteristics, health status, medical history, biological status, and dietary intake); and (2) standardized physical examinations (e.g., height, weight, and waist circumstance) and laboratory tests (e.g., blood and urine tests) [26]. We used 5-year aggregated cross-sectional data obtained by health interviews, physical examinations, and laboratory tests.

### Study population

**Fig. 1:**
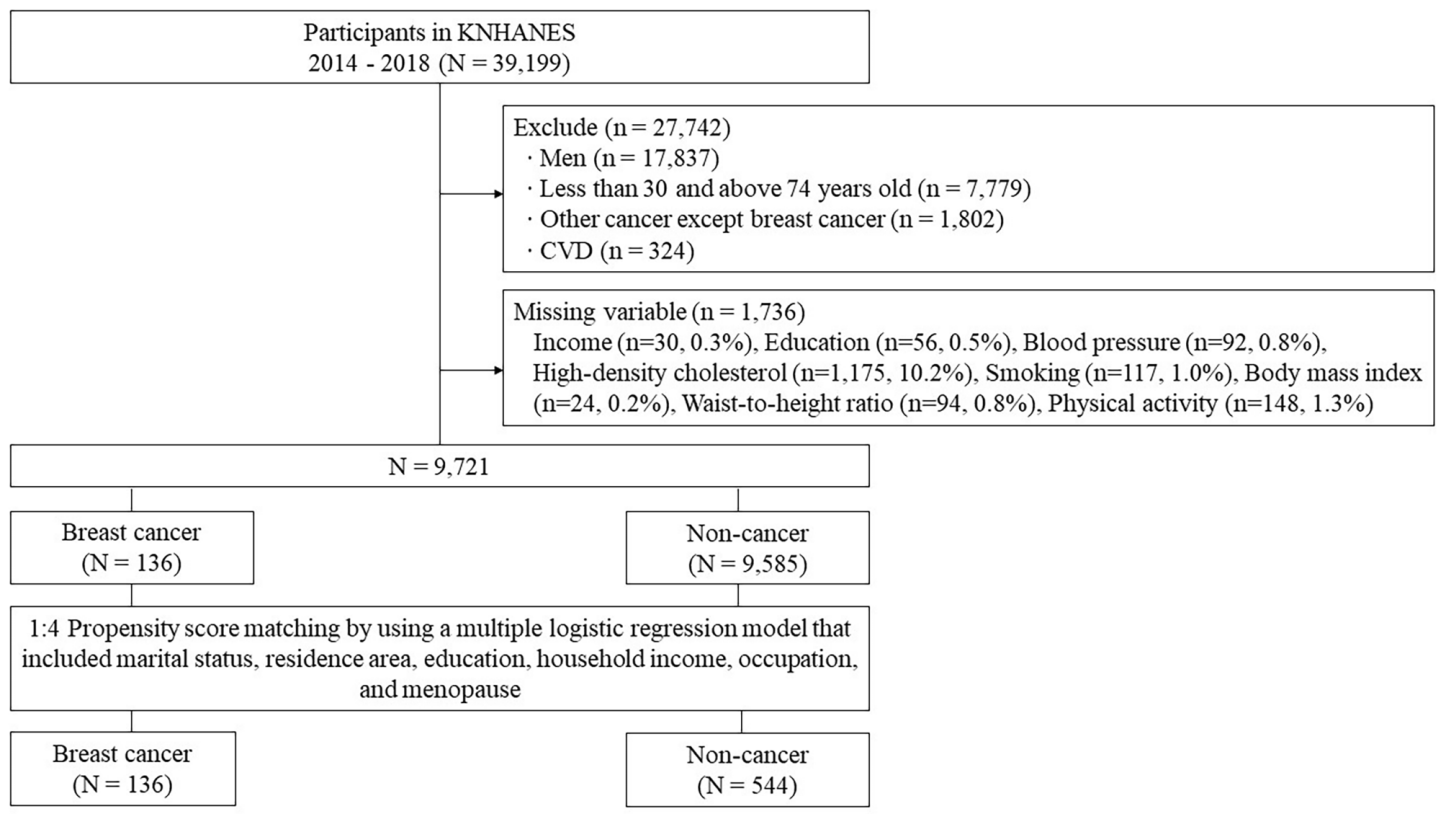
Participant inclusion flow chart

The process of sample selection was summarized in Fig. 1. Of 39,199 participants in the 2014–2018 KNHANES, we excluded 27,742 individuals who were men, less than 30 years or above 74 years old, diagnosed with other cancer, except for breast cancer, and diagnosed with CVD. To be consistent with the criteria used in the FRS system, we limited the age of women to 30–74 years and defined CVD as coronary heart disease, heart failure, peripheral artery disease, and stroke [22]. There was no breast cancer case in the 30 years or younger age group within the entire 2014–2018 KNHANES cohort. Moreover, we eliminated 1,736 individuals with missing variables, including income and education, blood pressure, cholesterol, anthropometric measures, smoking and physical activity (Fig. 1). From 9,721 remaining participants, 136 women had breast cancer and 9,585 were cancer-free.

**Fig. 2:**
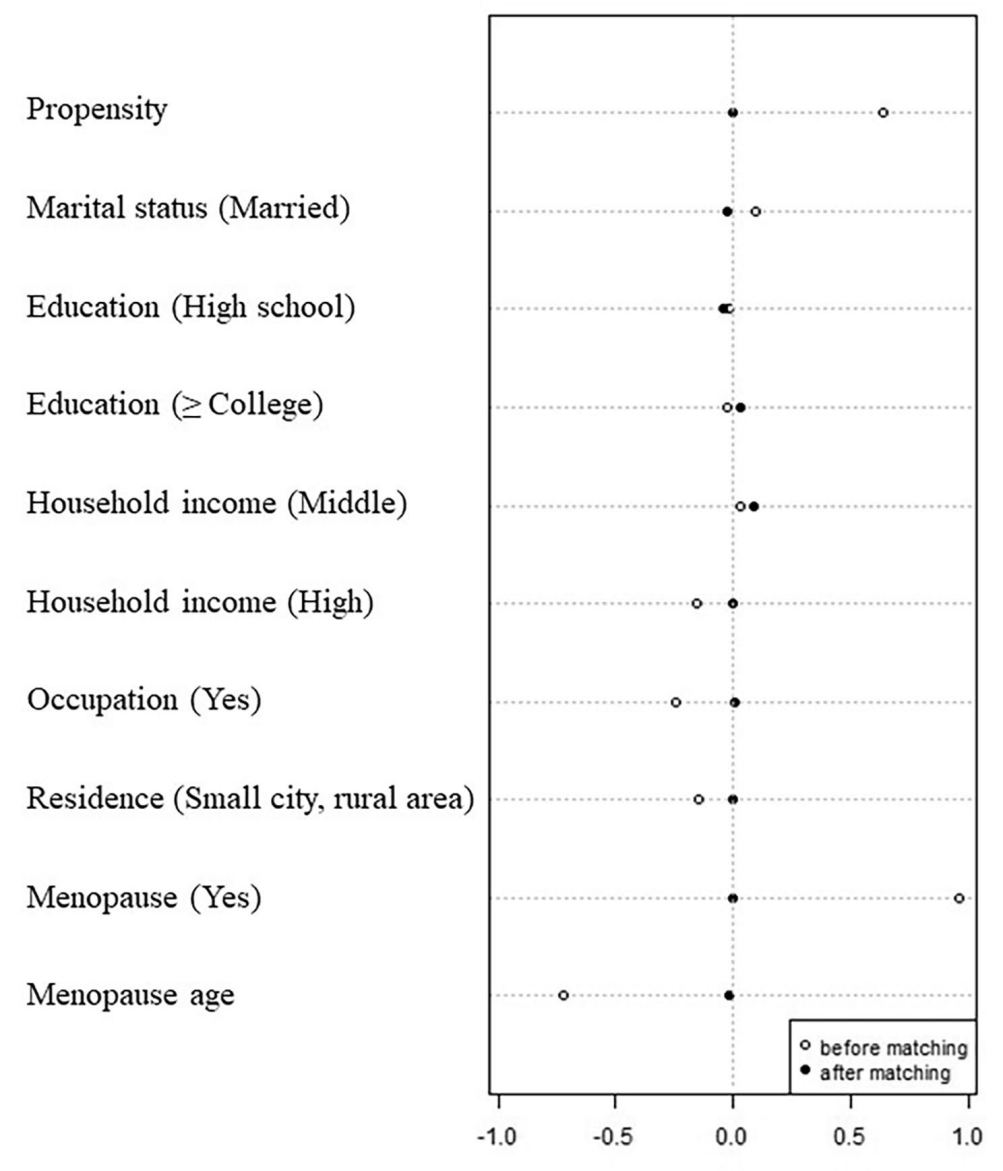
Love plot for baseline characteristics before and after propensity score matching

To assure balance between two groups, propensity score matching based on breast cancer diagnosis was performed using a 1:4 nearest-neighbor matching algorithm without caliper restriction [27, 28]. Due to a small number of breast cancer cases, we selected 1 to many (1:4) matching without caliper restriction to include more samples in the analysis. Standardized mean differences for the following variables were calculated before and after matching: marital status, education, household income, occupation, residence area, presence or absence of menopause, and menopause age. After propensity score matching, the included variables were well balanced with less than 0.1 standardized mean differences between groups (Fig. 2). For this study, the final sample comprises a total of 680 women, including 136 women with breast cancer and 544 women without cancer.

### Cardiovascular disease risk: framingham risk score

The FRS-assessed CVD risk was the outcome of the study. The FRS is a scoring system based on multiple traditional risk factors, including sex, age, total cholesterol, high-density cholesterol (HDL), systolic blood pressure, diabetes, and current smoking status. This composite score system was developed from the Framingham Heart Study cohort of adults aged 30–74 years with no CVD at baseline, conducted in the United States since 1948 [22]. FRS has been widely used in research and practice as an index to predict the CVD risk among adults of age 30–74 years as well as for specific subgroups, such as cancer survivors [12]. It predicts the risk of CVD events (i.e., coronary heart disease, angina, coronary revascularization, heart failure, peripheral artery disease, and stroke) within the next 10 years. The 10-year risk score can be derived as a percentage, and the risk is considered low for FRS “< 10%”, moderate for FRS “10–19%”, and high for FRS “≥ 20%” [22]. In this study, the FRS was categorized as “< 10%” (i.e., low risk) and “≥ 10%” (i.e., moderate/high risk), as only 2.2% (n = 15) of the total participants had FRS “≥ 20%”.

### Anthropometric measures of adiposity

Adiposity-related measures included BMI, waist circumference (WC), and WHtR based on current weight, height, and WC data obtained by physical examinations. BMI was calculated as weight (in kilograms) divided by height (in meters) squared (kg/m^2^), indicating the degree of overall adiposity. By the Asia-Pacific classification [29], BMI was categorized into 4 groups for this study: underweight (< 18.5 kg/m^2^), normal (18.5–22.9 kg/m^2^), overweight (23–24.9 kg/m^2^), and obese (≥ 25 kg/m^2^). Whereas BMI does not reflect the distribution of adipose tissue [18, 19, 30], WC (in centimeters) indicates abdominal adiposity, and the cutoff point of 80 cm was used by the Asia-Pacific guideline [29]. WHtR was calculated as WC (cm) divided by height (cm). Higher WHtR values indicate higher levels of abdominal adiposity, and the cutoff point was defined as 0.5 by previous research in Asian populations [31–33]. However, these cutoff points used for the study were not validated for women with breast cancer in Korea.

Asians, who have a shorter stature compared to Caucasians, are predisposed to develop visceral or abdominal adiposity, which leads to a higher prevalence of CVD risk factors at a lower BMI in Asians as compared to Caucasians [34–36]. The use of WC alone may underestimate the risk in individuals who are short, as short individuals with WC at a specified cutoff point may have more abdominal fat than tall individuals with the same WC [37]. Several studies, particularly among Asian populations, have reported that WHtR is a better predictor of the presence of CVD risk factors compared to other anthropometric measures such as BMI and WC [32, 38–40]. WHtR is more strongly associated with cardiometabolic risks than other anthropometric measures, particularly in non-obese populations [41, 42]. Considering the current evidence, we have focused on using WHtR as a more useful measure of abdominal adiposity in Korean women.

### Physical activity and sedentary time

The Global Physical Activity Questionnaire was used to assess physical activity calculated as the metabolic equivalent of task (MET)-minute per week (MET-min/week) by combining the intensity and time of physical activity during work, transport, and leisure activities based on the “usual week” [43]. Sedentary time was calculated based on responses to the question, “How many hours do you sit or lie down, excluding sleeping time?” A higher sedentary time represents more time sitting or lying down, categorized as “< 420 min/day” and “≥ 420 min/day” [20, 43].

### Other characteristics

Health-related characteristics included breast cancer diagnosis, time since cancer diagnosis, menopause-related information, alcohol intake, smoking status, diabetes, and hypertension by self-report. The menstruation information included self-reported presence or absence of menopause (yes or no), and age at menopause, if relevant. Menopause was defined as the cessation of menstruation for more than 12 months [44]. Survival time in years since a breast cancer diagnosis was calculated by subtracting self-reported age at diagnosis from the current age, and categorized into two groups: < 5 years as short-term and ≥ 5 years as long-term survival. In cancer literature, the 5-year survival benchmark is traditionally used for cancer patients, as most patients who do not have a recurrence during this period have a relatively low risk of recurrence [45].

Sociodemographic characteristics included age, marital status, education, household income, occupation, and residence area. Age was calculated by subtracting the birth year from the survey year. Household income was assessed by quantile using national standards (low, middle-low, middle-high, and high). For our analysis, we regrouped household income into 3 categories (low, middle, and high) after collapsing the categories of middle-low and middle-high into middle. Occupation (yes or no) was used to define whether the participant was employed or not.

### Statistical analyses

All analyses were performed using IBM SPSS Statistics for Windows version 25 (IBM Corp., Armonk, N.Y., USA). The study outcome, FRS, was compared after propensity score matching as we aimed to estimate the average treatment effect in the treated/exposed [ATT] (i.e., breast cancer). The propensity score was estimated using a logistic regression model in which breast cancer status regressed on the following variables: marital status, education, household income, occupation, residence area, presence or absence of menopause, and menopause age. Participants’ current age was not entered for estimating propensity scores because age was already included to calculate a composite FRS. The nearest-neighbor matching algorithm without caliper restriction was employed to form pairs of breast cancer and non-cancer participants (1:4 match). The balance after matching was evaluated using standardized mean differences. Baseline characteristics were considered well balanced between two groups if the standardized mean differences were less than 0.1 [27] (Fig. 2).

Descriptive statistics were used for all the variables included. Characteristics were compared between the breast cancer and non-cancer groups using the independent *t*-test for continuous variables and the chi-square test for categorical variables before matching. Because this study involved one-to-many matching, a generalized estimating equation was used to compare continuous and categorical variables in paired data after matching. In the breast cancer group, 2 × 2 ANOVA was used to compare FRS by WHtR (< 0.5 vs. ≥ 0.5) and survival time since a breast cancer diagnosis (< 5 years vs. ≥ 5 years). Statistical significance was set a priori as *p* < 0.05.

## Results

### Sample characteristics

Table 1 presents characteristics of 680 participants after 1:4 matching of 136 women with breast cancer and 544 women with no cancer. Most participants were married, had high school or higher levels of education, and had middle or high household income. 85% of the study sample reported being post-menopausal and mean age of menopause was 48 years (range 32–60).

**Table 1:** Baseline characteristics between non-cancer and breast cancer groups after propensity score matching

Characteristic	Matched Participants (N = 680)		
	Non-cancer (*n* = 544)	Breast cancer (*n* = 136)	std. mean diff.
	*n* (%) or M ± SD	*n* (%) or M ± SD	
Marital status^†^			
Single (ref)	14 (2.6)	4 (2.9)	
Married	530 (97.4)	132 (97.1)	0.022
Education^†^			
≤Middle school (ref)	183 (33.6)	46 (33.8)	
High school	186 (34.2)	44 (32.4)	0.039
≥ College	175 (32.2)	46 (33.8)	0.035
Household Income ^†^			
Low (ref)	240 (44.1)	55 (40.4)	
Middle	100 (18.4)	30 (22.1)	0.088
High	204 (37.5)	51 (37.5)	0.000
Occupation^†^			
No (ref)	235 (43.2)	59 (43.4)	
Yes	309 (56.8)	77 (56.6)	0.004
Residence area^†^			
Metropolitan (ref)	272 (50.0)	68 (50.0)	
Small city, rural area	272 (50.0)	68 (50.0)	0.000
Menopause^†^			
Yes (ref: No)	464 (85.3)	116 (85.3)	0.000
Age (years)	48.09 ± 4.67	47.98 ± 4.74	0.014
FRS (%)	5.47 ± 4.96	4.92 ± 4.20	0.119

^†^ Propensity score matching variables

Abbreviation: ref, reference variable; std.diff., standardized difference; M, mean; SD, standard deviation; FRS, Framingham Risk Score

### Comparison of FRS and other CVD risk factors

Prior to matching, women with breast cancer (n = 136) were older with a mean age of 57 years (85% at menopause) compared to mean age of 51 (50% at menopause) in non-cancer women (n = 9,585) (*p* < 0.001). In the propensity score matched sample (n = 680), the mean age was 57 years old for both groups, ranging from 31 to 74 years. The average years since breast cancer diagnosis was 8.5 ± 7.0 (range 0–37 years), and 65% of women with breast cancer were identified as long-term survivors (≥ 5 years). Forty-one women (30%) reported to have lived with breast cancer for more than 10 years. Forty-eight women (35%) survived less than 5 years from their breast cancer diagnosis, including 12 women under active cancer treatment (i.e., survival < 2 years).

Prior to propensity score matching, women with breast cancer showed mean FRS of 4.9% compared to 4.1% in women with no cancer. After matching, similar mean FRS levels were reported as 4.9% (range 0.9–24.8%) in the breast cancer group and 5.5% (range 0.9–30.1%) in the non-cancer group (*p* = 0.153). Only approximately 16–17% of each group showed FRS ≥ 10%, categorized as moderate/high risk. The group with breast cancer showed significantly lower CVD risk levels in some individual components, such as higher HDL (*p* = 0.006) and lower total cholesterol (*p* = 0.034) than the non-cancer group. Hypertension was reported in 23.5% of breast cancer survivors vs. 25.4% of no cancer women. The percentages of those previously diagnosed with diabetes were 12.5% in breast cancer survivors vs. 11.8% in non-cancer women (Table 2).

We also assessed anthropometric measures of adiposity and physical activity, which were not accounted for by FRS calculation. Women with breast cancer had lower BMI (*p* = 0.031) and WHtR (*p* = 0.048) than women without cancer, but similar means of WC (*p* = 0.211). The two groups had similar levels in all physical activity variables, including total physical activity in MET-min/week and sedentary time in minutes per day.

**Table 2:** Comparison of Framingham Risk Scores and other cardiovascular disease risk factors after propensity-score matching (N = 680)

Characteristic	Non-cancer (*n* = 544)	Breast cancer (*n* = 136)	*p*-value
	*n* (%) or M ± SD	*n* (%) or M ± SD	
FRS (%)^†^	5.47 ± 4.96	4.92 ± 4.20	0.153
Low risk (< 10)	451 (82.9)	114 (83.8)	0.793
Moderate/high risk (≥ 10)	93 (17.1)	22 (16.2)	
Age (year)	57.15 ± 9.83	57.12 ± 9.60	0.957
SBP (mmHg)	119.70 ± 17.72	117.71 ± 17.74	0.223
DBP (mmHg)	75.17 ± 9.62	74.45 ± 8.91	0.393
HDL cholesterol (mg/dL)	53.39 ± 12.801	57.02 ± 14.46	0.006*
Total cholesterol (mg/dL)	201.23 ± 38.12	193.71 ± 39.30	0.034*
Diabetes (yes)	64 (11.8)	17 (12.5)	0.821
Hypertension (yes)	138 (25.4)	32 (23.5)	0.653
Current smoker (yes)	29 (5.3)	2 (1.5)	0.076
Alcohol intake (times/week)			
< 2	496 (91.2)	127 (93.4)	0.414
≥ 2	48 (8.8)	9 (6.6)	
BMI (kg/m^2^)	23.86 ± 3.28	23.18 ± 3.14	0.031*
< 18.5 (underweight)	14 (2.6)	5 (3.7)	0.023*
18.5–22.9 (normal)	219 (40.3)	66 (48.5)	
23-24.9 (overweight)	139 (25.6)	35 (25.7)	
≥ 25 (obese)	172 (31.5)	30 (22.1)	
Waist Circumference (cm)	80.61 ± 8.74	79.49 ± 9.17	0.211
< 80	263 (48.3)	77 (56.6)	0.088
≥ 80	281 (51.7)	59 (43.4)	
Waist to Height Ratio (WHtR)	0.52 ± 0.06	0.51 ± 0.06	0.048*
< 0.5	210 (38.6)	63 (46.3)	0.094
≥ 0.5	334 (61.4)	73 (53.7)	
Total Physical Activity (MET-min/week)	807.78 ± 1287.98	807.94 ± 1000.94	0.999
Work			
Vigorous	10.81 ± 207.45	10.59 ± 123.48	0.987
Moderate	90.81 ± 665.64	29.12 ± 131.86	0.087
Leisure			
Vigorous	68.01 ± 333.58	97.94 ± 489.65	0.503
Moderate	161.73 ± 383.80	204.41 ± 473.61	0.324
Transport	476.42 ± 731.15	465.88 ± 707.34	0.873
Sedentary Time (min/day)	459.39 ± 203.89	436.40 ± 208.38	0.243
< 420	230 (42.3)	70 (51.5)	0.050
≥ 420	314 (57.7)	66 (48.5)	

^†^ Framingham Risk Score (age, SBP, HDL cholesterol, total cholesterol, diabetes, current smoker)

Abbreviation: BMI, body mass index; DBP, diastolic blood pressure; HDL, high-density lipoprotein cholesterol; LDL, low-density lipoprotein cholesterol; SBP, systolic blood pressure

*p < 0.05

Three measures of adiposity, BMI, WC, and WHtR were highly correlated with each other in both groups (*r *values ≥ 0.85, *p* values < 0.01). Among these measures, WHtR showed the strongest positive correlation with FRS in both groups (*r* = 0.54 for breast cancer and *r* = 0.42 for no cancer). Table 3 presents the FRS in women with breast cancer by WHtR (< 0.5 vs. ≥ 0.5) and the time since breast cancer diagnosis (< 5 years vs. ≥ 5 years). 2 × 2 ANOVA revealed a significant association of WHtR with FRS in that breast cancer survivors with WHtR ≥ 0.5 had higher FRS than those with WHtR < 0.5 [*F* (1, 132) = 24.217, *p* < 0.001, partial *η*^*2*^ = 0.155]. However, FRS was not associated with survival years since breast cancer diagnosis [*F* (1, 132) = 2.989, *p* = 0.086, partial *η*^*2*^ = 0.022].

**Table 3:** Framingham Risk Scores by waist-to-height ratio and time since cancer diagnosis in women with breast cancer (*n* = 136)

	FRS (M ± SD)	
	WHtR < 0.5	WHtR ≥ 0.5
Time since cancer diagnosis < 5years	2.52 ± 1.97 (n = 27)	5.48 ± 3.67 (n = 21)
Time since cancer diagnosis ≥ 5 years	3.29 ± 2.62 (n = 36)	7.07 ± 4.99 (n = 52)

Abbreviation: FRS, Framingham Risk Score; WHtR, Waist to Height Ratio

## Discussion

This study aimed to investigate FRS-based CVD risk in Korean women with breast cancer compared to a propensity-score matched group of women without cancer. Using cross-sectional data, we hypothesized that Korean women with breast cancer would have worse FRS, indicating a higher risk of future CVD than women with no cancer. However, our results revealed no significant difference in FRS between the two groups. Surprisingly, the breast cancer group presented a significantly lower CVD risk with lower total cholesterol, BMI, and WHtR and higher HDL than the non-cancer comparison group. These findings suggest important insights into the CVD risk profiles of Korean women with breast cancer and highlight the need for continued CVD risk management in this population.

Our finding using the 2014–2018 KNHANES database may be attributed to the significant clinical benefits of advances in cardioprotective practices in cancer care over the last decades, including reduced anthracycline use and modern radiotherapy techniques to minimize radiation exposure to the heart [46–49]. According to a recent epidemiology study in the United States that involved over 500,000 breast cancer survivors from 1975 to 2017, there was a trend of significant decline in CVD mortality in breast cancer survivors compared to the general population [50]. Furthermore, the age of initial breast cancer diagnosis became much younger in Korea (median age of 52 years) compared with women in Western countries (median age of 62 years) [51, 52]. Younger Korean breast cancer survivors in this study (mean age of 57 years) were likely to have a low burden of CVD risk [51]. Additionally, all Korean citizens have access to universal health care services through government-managed health insurance and are encouraged to screen both breast cancer and CVD [53, 54]. Korea is one of the countries (e.g., Norway, United Kingdom, etc.) reporting a declining trend of age-standardized CVD mortality over the past 35 years [55]. We speculate that national-level support for the prevention of and screening for CVD risks might have contributed to such results.

Our findings add new insights to the extant literature on CVD risk in Korean breast cancer survivors. In our sample, based upon the FRS, most women were at the low-risk category (FRS < 10%). Our results contradict to the results of another Korean study with the KNHANES 2007–2013 data including both men and women cancer survivors. Much higher percentages in FRS were reported as 19.1% in cancer survivors and 13.3% in non-cancer controls [12]. In their study, the mean FRS percentages in women with cancer and non-cancer women were 12.5% and 6.7%, respectively [12]. Particularly, patients with hepatic, colon, lung, breast, and gastric cancer had higher FRS [12]. Not surprisingly, cancer survivors were much older than non-cancer controls, mean age of 60 and 45 years, respectively [12]. However, the findings were based upon univariate analysis without adjusting significant differences in baseline sociodemographic factors between the cancer and non-cancer groups [12]. Similarly, in our initial study sample prior to matching, most breast cancer survivors were at menopause (mean age of 57) compared to only the half of women with no cancer at menopause (mean age of 51). To balance differences in baseline characteristics, we generated propensity scores to estimate the ATT based on breast cancer diagnosis. Nearest-neighbor matching at 1:4 ratio without caliper restriction was used to generate the comparison group. The propensity scores matched breast cancer and non-cancer groups with mean age of 57 years showed similar FRS, indicating no association of breast cancer with CVD risk in Korean women.

Women with breast cancer in the present study displayed fewer current smokers and diabetes and lower adiposity and FRS levels compared to the previous Korean study [12]. However, total cholesterol was much higher in our study sample of women (breast cancer group, 193 mg/dL; non-cancer group 201 mg/dL) compared to the levels reported by So et al. (cancer group, 187 mg/dL, non-cancer group 188 mg/dL) [12]. Another concerning finding in our sample is the high cholesterol levels that indicate borderline cardiometabolic risk in postmenopausal women in both groups, regardless of their cancer diagnosis (85% prevalence in both groups). Given the lack of public awareness of CVD risk in postmenopausal women [55] and suboptimal management of CVD in breast cancer survivors [56, 57], our findings indicate the importance of proactive screening and management of CVD risk factors (e.g., periodical lipid checkups) in postmenopausal women, including breast cancer survivors.

The use of FRS in assessing CVD risk for breast cancer survivors has not been properly validated, and no standardized guideline is currently available. The FRS, a validated tool for assessing global CVD risk, was originally derived from white men of European descent [58]. Several studies have suggested that the FRS may overestimate the CVD risk in Asian populations, including Koreans [59, 60]. A recent nationally representative prospective cohort study in Korea showed that the FRS overestimated 3–6 times as many coronary heart disease events as were observed [60]. On the other hand, according to a study with 152 breast cancer survivors at a cardiology-oncology clinic in Canada, Law et al. [58] reported that the FRS underestimated the true incidence of future CVD in over forty months median follow-up in their sample. Whereas over half of the breast cancer survivors were in FRS categories of moderate- or high-risk, breast cancer survivors who were considered being at low-risk by FRS still experienced cardiotoxicity from breast cancer treatment [58, 61]. These findings suggest that even breast cancer survivors with low CVD risk by FRS may still require close monitoring over their extended periods of survivorship to detect long-term cardiotoxic effects of cancer treatment [58, 61]. Since FRS does not account for lifestyle changes, such as abdominal adiposity, resulting from breast cancer diagnosis and treatment, it is important to exercise caution when using FRS for breast cancer survivors in Korea [6–8].

Similar to the general population, adipose tissue distribution, specifically abdominal/visceral adiposity, is a stronger predictor of CVD and overall mortality in breast cancer survivors than overall adiposity measured by BMI [9, 23, 24]. An increased CVD risk was associated with only high BMI levels ≥ 35 kg/m^2^ (i.e., obese Class II) in breast cancer survivors, and 11% of normal-weight women had excess adiposity and reclassified as high CVD risk [9]. Asians have a higher percentage of body fat compared to Western populations at a lower BMI and WC [62, 63]. In our study sample of women with breast cancer, the average BMI was 23.2 with only 22% of BMI ≥ 25 kg/m^2^ but about 54% had WHtR ≥ 0.5, indicating elevated levels of abdominal adiposity at a normal BMI. Several studies, particularly among Asian populations, have reported that WHtR is a better predictor of CVD risk factors compared to other anthropometric measures [41, 42]. Our findings suggest that WHtR plays a significant role in FRS-based CVD risk among breast cancer survivors, irrespective of their survival years after the cancer diagnosis. Although there is currently no consensus yet on the clinical utility of WHtR over other traditional measures, it appears to be a more useful tool for assessing abdominal adiposity in Asians than non-Asians [64]. Large-scale cohort studies are needed to explore the role of WHtR in predicting long-term CVD risks in Korean women with breast cancer.

This secondary data analysis has several limitations. First, KNHANES was not specifically designed to evaluate breast cancer survivorship, which may limit the ability to generalize our results to all breast cancer survivors in Korea. Secondly, the information on breast cancer diagnosis and treatment was unavailable, which limited further analyses related to any long-term treatment-related cardiotoxicity. Survival time was the only information we were able to use from the given data. Speculating from recent reports [50, 53], most in this study may have been early-stage, hormonal-sensitive breast cancer, treated with surgery and adjuvant therapy with cardioprotective strategies. The cross-sectional study design limited our understanding of trends in characteristics of breast cancer patients and cardio-oncology practices in Korea during their extended survivorship. While the study outcome, FRS, is a useful tool for assessing the global risk of future CVD events, it does not represent a true CVD endpoint. Lastly, medical histories and health behaviors were collected by self-reports based on simplistic measures (e.g., menopause status and physical activity), compromising the accuracy of the data due to recall and/or misclassification biases and potential measurement errors.

## Conclusion

Our study found no association between breast cancer and FRS-based CVD risks, suggesting similar 10-year CVD risk levels between Korean women with and without breast cancer, who had comparable baseline characteristics determined by propensity score matching. Our findings contradict previous studies and suggest that women with breast cancer had a lower risk category of FRS with lower levels of abdominal adiposity and lipids compared to their counterparts. Potential explanation for these results may include the continued efforts towards cardio-protection during cancer treatment, as well as the governmental-managed health care system in Korea. Importantly, these mostly postmenopausal, women need proactive screening and management for CVD risk factors, such as regular lipid and adiposity checkups, regardless of their breast cancer history. As breast cancer and CVD vary across different geographic regions and racial/ethnic populations, future studies are needed to investigate the trajectories of modifiable risk factors for CVD and CVD hard endpoints among breast cancer survivors in Korea.

## Data Availability

The original study data (2014–2018 KNHANES Basic DB) are publicly available for free on the KNHANES website (https://knhanes.kdca.go.kr/knhanes/sub03/sub03_02_05.do) with no restriction apply to the availability of these data.

## List of Abbreviations

ATT: Average treatment effect in the treated
BMI: Body mass index
CVD: Cardiovascular disease
FRS: Framingham Risk Score
GPAQ: Global Physical Activity Questionnaire
HDL: High-density cholesterol
KNHANES: Korea National Health and Nutrition Examination Survey
MET: Metabolic equivalent of task
WC: Waist circumference
WHtR: Waist-to-Height Ratio
